# Vaccinia Virus: From Crude Smallpox Vaccines to Elaborate Viral Vector Vaccine Design

**DOI:** 10.3390/biomedicines9121780

**Published:** 2021-11-26

**Authors:** Onur Kaynarcalidan, Sara Moreno Mascaraque, Ingo Drexler

**Affiliations:** Institute for Virology, Düsseldorf University Hospital, Heinrich-Heine-University, 40225 Düsseldorf, Germany; onurkaynarcali22@hotmail.com (O.K.); Sara.MorenoMascaraque@med.uni-duesseldorf.de (S.M.M.)

**Keywords:** cross-protection, poxvirus, VACV, recombinant MVA, genetic engineering, immune-evasive genes, host-range-related genes, viral vector vaccines

## Abstract

Various vaccinia virus (VACV) strains were applied during the smallpox vaccination campaign to eradicate the variola virus worldwide. After the eradication of smallpox, VACV gained popularity as a viral vector thanks to increasing innovations in genetic engineering and vaccine technology. Some VACV strains have been extensively used to develop vaccine candidates against various diseases. Modified vaccinia virus Ankara (MVA) is a VACV vaccine strain that offers several advantages for the development of recombinant vaccine candidates. In addition to various host-restriction genes, MVA lacks several immunomodulatory genes of which some have proven to be quite efficient in skewing the immune response in an unfavorable way to control infection in the host. Studies to manipulate these genes aim to optimize the immunogenicity and safety of MVA-based viral vector vaccine candidates. Here we summarize the history and further work with VACV as a vaccine and present in detail the genetic manipulations within the MVA genome to improve its immunogenicity and safety as a viral vector vaccine.

## 1. Introduction

Orthopoxviruses are large, enveloped, double-stranded DNA (dsDNA) viruses that belong to the large family of *Poxviridae*. This family is able to harbor large foreign DNA sequences within their genome, making them ideal vector systems to express heterologous antigens from various pathogens [1]. Additionally, poxviruses replicate within special organelles which are established after infection in the cytoplasm called viral factories rather than the host nucleus [2,3,4] which greatly reduces the possibility of viral DNA integration into the host genome preventing the interruption of host genes and carcinogenesis. They can infect a wide variety of cell types, including keratinocytes, fibroblasts, and immune cells such as antigen-presenting cells [5]. Recombinant vaccinia viruses (VACV) are easy to generate, genomically stable, and can induce long-term cellular and humoral immune responses [6]. Today, recombinant vaccines based on various strains of poxviruses are in different phases of clinical trials [1].

VACV, a member of the poxvirus family, has been extensively used to induce cross-protection against variola virus (VARV), thanks to highly conserved structural proteins with other orthopoxviruses (OPXV), reduced virulence, and high immunogenicity [7,8]. On 8 May 1980, the 33rd World Health Assembly announced that VARV (smallpox) was eradicated worldwide as a result of their global vaccination campaign [9]. OPXVs have highly conserved structural proteins, resulting in cross-protective reactivity. Therefore, vaccination with one OPXV induces the production of cross-neutralizing antibodies that prevent infection with other OPXV strains [10]. Various VACV strains have been developed and used in different countries during the global smallpox eradication campaign prior to standardized vaccination procedures and quality control methods [11,12]. The most commonly used strains were New York City Board of Health (NYCBH) in North America, Tian Tan (VTT) in China, Dairen and Ikeda in Japan, Bordeaux and Chambon for Africa, and finally, Lister, modified vaccinia virus Ankara (MVA), Bern and Paris in Europe [13,14]. After the eradication of smallpox, VACV (as well as its attenuated strains) are still popular in efforts to develop novel prophylactic or therapeutic recombinant vaccines against infectious or neurological diseases e.g., Alzheimer’s disease [15]. The first recombinant VACV vaccine candidate was developed against Hepatitis B in 1982 [16]. Since then, VACV has been used to create recombinant vaccines against rabies [17], HIV [18,19], and many other pathogens plaguing humanity.

The ease to manipulate the VACV genome and recombine foreign DNA with it has advanced research in several scientific fields such as immunology and virology [13]. In this review, we will discuss the history and modern methodologies to generate VACV-based vaccines, as well as recent approaches to optimizing VACV vaccine candidates. 

## 2. The Origin and History of Smallpox and First Smallpox Vaccination

Smallpox, caused by variola virus (VARV) major and minor [11], is an acute contagious disease that caused several hundred million deaths and devastated civilizations around the world. VARV is believed to have initially infected humans around 10,000 BCE in the early agricultural settlements of northeastern Africa [18,19] and was introduced to Europe between 400 and 600 CE [20]. Records from China in 1122 BCE and India around 1500 BCE contain descriptions that suggest smallpox or a smallpox-like disease [21] and skin lesions resembling those of smallpox were found on the mummified head of Egyptian pharaoh Ramses V [22]. In 2016, researchers isolated and reconstructed a draft genome of an antique strain of VARV from the mummy of a child who lived in Lithuania around 1650 CE. Typically, some VARV genes are fragmented and non-functional, while their homologs in VACV are sound. Comparison of these sequences to VACV showed that this elapsed VARV strain contained the same gene fragmentation pattern as modern VARV strains from the 20th century suggesting that the functional loss took place before 1650 CE [20]. More recently, VARV sequences were discovered and isolated from remains from 600 CE in Northern Europe that expose the existence of a now-extinct VARV clade [23,24]. 

All species of OPXV are morphologically similar and VARV major and minor are only distinguishable by real-time polymerase chain reaction (RT-PCR). Despite their similarities, they differ greatly in disease severity with case-fatality rates of 1% for variola minor and 30% for variola major [25]. While the origin of VARV and the first smallpox case in history are still uncertain, taterapox virus (TATV), which replicates in rodents, is the closest known relative of VARV. The WHO hypothesizes that rodents infected humans with an African rodent-borne variola-like or proto-variola virus about 16,000–68,000 years ago that eventually evolved into the human smallpox causing VARV [21,26].

Before vaccination was introduced in the late 1700s, variolation was used to protect individuals from smallpox. Variolation is a procedure in which healthy individuals are inoculated with powder from pustules or scabs of infected individuals who developed fewer clinical signs and symptoms in order to induce protection against smallpox infection [11]. Variolation had a significantly decreased mortality rate of 2–3%, compared to smallpox with a 15–30% mortality rate [27]. However, variolated people could transmit smallpox, particularly if the variolation site was left uncovered. This led to new outbreaks and made it clear that a better solution was sorely needed.

In the second half of the 18th century, it was frequently observed that milkmaids who acquired the commonly named cowpox disease were protected against smallpox disease [11]. Similarly, Edward Jenner and others observed that the inoculation with infection material from blisters of infected cows or infected milkmaids protected humans against smallpox [28]. Although Edward Jenner may not have been the first to practice this method, he was the first to publish it [29]. Instead of pus taken from VARV lesions, he inoculated individuals with cowpox pus initially taken from a skin lesion on a milkmaid’s hand. This procedure was later called vaccination. James Phipps, the first recipient of Edward Jenner’s vaccine, did not develop any sign of disease after a challenge with smallpox and this was confirmed when sixteen other vaccinated individuals were protected against smallpox exposure. This was an important scientific landmark in the history of vaccination [30]. In the absence of any research ethics committee, Jenner used human subjects, including children, in his research [31].

**Figure 1:**
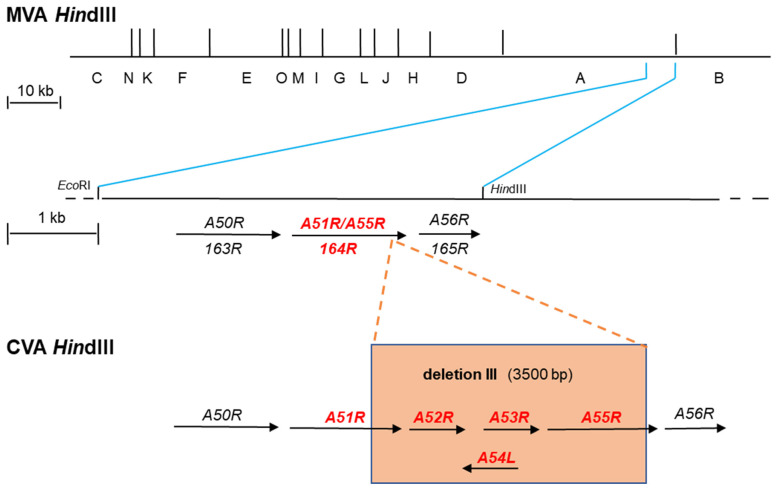
Illustration of the MVA genome and comparison of two distinct gene nomenclature systems which are currently applied. The VACV strain Copenhagen nomenclature relies on digestion of the viral genome using the restriction enzyme *Hi*ndIII. In the resulting *Hind*III map, the fragments obtained are alphabetically labeled from A to O. The largest fragment is named A and the smallest as O. Since the genome is bidirectional, genes are labeled R (right) and L (left) to indicate the genomic orientation of ORFs. The *Hind*III map of the MVA genome is shown (MVA *Hin*dIII). During attenuation in cell culture, chorionallantios vaccinia virus Ankara (CVA) the parental strain of MVA, suffered amongst others six major deletions within its genome resulting in strain MVA. Deletion III is shown as an example (orange box) (CVA *Hin*dIII). This deletion is located at the right end of the viral genome. In MVA, the flanking regions of deletion III are depicted within a fragment obtained when the *Hin*dIII A fragment is cut with *Eco*RI. In strain Copenhagen and strain CVA this fragment harbors amongst others the genes *A50R* to *A56R*. In contrast, deletion III resulted in the loss of genes *A52R* to *A54L*, truncation of *A51R*, and fragmentation of *A55R* (orange box). The latter two genes are expressed as a fusion gene (*A51R/A55R*) of unknown function in MVA. Another more recent nomenclature is based on sequencing data of the full genome of MVA (177,923 bp) coding sequences are numbered according to their first appearance starting from the left to the right end of the genome. The first ORF present 5’ in the genome is termed *001* and labeled R or L to indicate the genomic orientation. A more detailed illustration of the MVA genomic part after the occurrence of deletion III is shown (MVA *Hin*dIII). The genomic sequence from 146,084 bp (*Eco*RI) to 150,758 bp (*Hin*dIII) corresponds to the part obtained after cutting the *Hin*dIII A fragment with *Eco*RI. According to this, the nomenclature neighboring genes are hierarchically labeled including the gene *164R* which corresponds to the *A51R/A55R* fusion gene in the Copenhagen nomenclature.

There is growing evidence that Jenner was actually working with a poxvirus of equine origin that was transmitted from horses to cows instead of the cowpox virus (CPXV) [32]. In 1939, the smallpox vaccines were shown to be distinct from CPXV and were subsequently named VACV [33,34]. VACV is distinct from CPXV and VARV as it is more closely related to horsepox. However, the origin of VACV is currently unknown [13,35]. The VACV genome size is approximately 190 kbp encoding over 200 open reading frames (ORFs) [36]. The genome is bidirectional and genes are labeled R (right) and L (left) to indicate the genomic orientation of ORFs. Historically the genomes have been digested with restriction enzymes for further investigation. After digesting the genomes using *Hin*dIII the resulting 15 fragments which were obtained were specified alphabetically. Thereby, the largest fragment received the letter A and the smallest O [37,38] (Figure 1). While the historical *Hin*dIII fragment letter/ORF number nomenclature was based on the VACV strain Copenhagen [39], improved sequencing techniques and bioinformatic analysis were applied in the following years that resulted in a distinct ORF-based nomenclature for other strains such as Western Reserve (WR) [40] and MVA [41]. Based on the sequencing data, the genomes were screened for the presence of ORFs starting from the left to the right end of the genome. ORFs were numbered according to their first appearance. The first ORF found 5’ in the genome was termed 001, e.g., *VACWR001* for the first ORF in the WR genome. Additionally, the ORFs were labeled R or L to indicate the genomic orientation in MVA, e.g., *MVA001L* for the first ORF in the genome with orientation to the left end (Figure 1 and Table 1).

A first wave of smallpox vaccines using VACV was employed during the global smallpox eradication campaign from 1967 to 1977. The majority of vaccines used in this program were manufactured using live animals [51]. In order to minimize microbial contamination and allergic reactions upon vaccination, second-generation VACV vaccines were further produced in tissue culture systems or in embryonated chicken eggs [14]. Due to adverse effects from first- and second-generation VACV vaccines, it was necessary to improve the safety of the vaccines. In order to achieve this, the third generation VACV was obtained by passaging wild-type VACV in alternative hosts via cell culture [13]. Some examples of third-generation vaccines tested as vaccine candidates include Lister clone 16m8 (LC16m8), Dairen I strain (DIs), MVA, and several attenuated avipoxviruses [12].

Over the past four decades, VACV has been developed as a recombinant viral vector and vaccine. As a consequence of novel bioengineering techniques, the deletion, as well as reintroduction of immunomodulatory and host range genes, has been used to optimize the immunogenicity and safety of poxvirus-based vaccines. These genetically modified VACV strains, most of them completely sequenced, have been used to create improved vaccines [1]. 

## 3. Live Animal-Derived Vaccines and the Eradication of Smallpox

Devastating epidemics of smallpox left a deep impression on society, as they were associated with extremely high rates of mortality. When there was an outbreak of smallpox in small populations, such as the early colonial settlements in North America or Iceland, the disease would subsequently die out due to a lack of suitable hosts. After Jenner laid the foundation of what would become modern vaccinology, vaccination spread rapidly and was soon successfully combating the smallpox epidemic. By the end of the 20th century, smallpox was on its way to eradication [11].

After the World Health Organization (WHO) was founded in 1948 and United Nations Children’s Funds (UNICEF) in 1946, vaccine programs were mounted throughout the world [52]. However, it was not until the 1960s that reliable assays and protocols for quality control of these vaccines were established [14]. In 1966, the global fight against smallpox intensified with the ultimate goal of globally eradicating smallpox. The program was based upon massive VACV-based vaccination campaigns and surveillance systems to detect and contain outbreaks [13]. The WHO initiated the Expanded Programme on Immunization (EPI) in 1974, which aimed to drastically increase vaccination rates in children from developing countries [52]. No particular parental VACV strain was officially recommended by WHO [14], resulting in the use of various strains during the program (such as New York City Board of Health, EM-63, or Lister), with a vast majority manufactured on the skin of live animals such as calves, buffaloes, sheep, or rabbits [13].

Although the last case of naturally acquired smallpox occurred in Somalia in 1977, Janet Parker was the last person to die of smallpox in 1978 in Birmingham, England. She was infected at the city’s University Medical School where they conducted smallpox research. She likely contracted the virus via an airborne route through the building’s duct system or direct contact while visiting the microbiology corridor, illustrating the extraordinary contagiousness of smallpox and the necessity of appropriate laboratory safety measures [53,54,55]. Due to continued efforts to eradicate smallpox and the fact that humans represent the only known natural VARV reservoir, the 33rd World Health Assembly, the decision-making body of WHO, finally declared the disease eradicated on 8 May 1980. Today, VARV is retained at two WHO collaborative centers, the Center for Disease Control and Prevention (Atlanta, USA) and the State Research Center of Virology and Biotechnology (Novosibirsk, Russia) [14].

## 4. Cell- or Tissue-Derived Smallpox Vaccines 

The use of live animals for the manufacturing of vaccines was essential for the development of first-generation smallpox vaccines. However, this generation had several issues including inadequate standardized procedures, quality control issues resulting in possible microbial contamination, and sensitization to allergenic animal proteins that remained in the vaccine after production. In order to address these issues, the next generation of vaccines was later manufactured in tissue culture systems or embryonated chicken eggs [13]. This vaccine generation is further characterized by controlled and optimized manufacturing practices, and minimal cell culture passages [56]. In addition, most of these vaccine strains were not cloned as single plaque isolates and genetic modifications in viral genomes happened, if at all, randomly and were not intended or further investigated. The first vaccine of this type of production was generated in 1960 by passaging the Lister strain either in rabbit kidney cells, primary cells derived from chicken embryos, or on chorioallantoic membranes of chicken embryos. In a different vaccine, the calf lymph Lister vaccine was directly transferred to cells without further passages. Both vaccines induced similar neutralizing antibody titers [11]. This cell-derived Lister vaccine showed no severe complications in clinical trials in Asia and Europe [14]. Other VACV vaccines that were generated using Lister as the parental strain, such as the Elstree-BN, were passaged in chicken embryo cells and manufactured for public use by Bavarian Nordic after preclinical studies in non-human primates yielded a safe and immunogenic profile [56,57,58]. Sanofi Pasteur developed the Lister/CEP (chicken embryo primary cell) vaccine by passaging the live animal-derived Lister vaccine three times in chicken embryo primary cells, which proved to have comparable safety and immunogenicity to the parental vaccine strain [59]. VACV vaccines that originate from the NYCBH strain had a rough start after the first candidate (grown in cell culture) failed to demonstrate an adequate take rate in 1968. The take is the protective immune reaction that restricts viral replication to the superficial skin layers in which the vaccine was inoculated via scarification with a bifurcated needle. For the vaccine to take, it must cause a visible skin lesion at the inoculation site with a minimum size of 1 cm in diameter in order to correlate with protection against smallpox [60]. Despite the early failure, this strain was further passaged in MRC-5 cells and used to generate the Cell Culture Smallpox Vaccine [14]. A different vaccine, ACAM2000® which is currently licensed in the USA was derived from Dryvax (an NYCBH strain) grown in Vero cells [57]. Another descendent of the NYCHB strain, WR, is a neurovirulent strain that has been widely passaged in rabbits, mice, and cell culture [14]. Further details on passaging and properties of cell- and tissue-derived vaccines have been reviewed elsewhere [14].

Cell- and tissue-derived smallpox vaccines can induce reactogenicity similar to that found in live animal-derived vaccines. Even though many of these vaccines have not been directly confronted by smallpox, they will likely retain the same efficacy as the live animal-derived vaccines due to their close relation [57].

Although live animal-derived vaccines widely lacked standardization and control, cell- and tissue-derived vaccines were strongly improved in this respect and more predictable due to the use of cell culture systems allowing upscaling in the manufacturing process and reduction in adverse events [56]. However, the use of replication-competent strains of VACV has its own set of risks [14]. This, along with a substantial minority of the populace having contraindications against the use of live animal or cell- or tissue-derived smallpox vaccines, emphasized the importance and need to develop a new vaccine generation that would be safer but still maintain an immunoprotective profile [13].

## 5. Smallpox Vaccines with Improved Safety Profile 

Since cell- or tissue-derived vaccines still showed an unsatisfactory safety profile [14], the following generation of VACV vaccines was generated with the objective to create highly attenuated or non-replicative VACV strains that retained their immunogenicity and protective capacity against smallpox [13]. These vaccines were likely initiated in the early 1970s as a response to severe side effects commonly caused by live animal-derived smallpox vaccines [57]. Particularly, adverse reactions due to systemic spreading of the vaccine virus were considered as serious when presented as they included (but were not limited to) generalized vaccinia, eczema vaccinatum, and post-vaccinial encephalitis. VACV was attenuated by extensively passaging the parental strains in tissue cell cultures from various hosts [14]. This led to the alteration of viral properties through random genomic mutations and deletions that included, but were not limited to, genome composition, virulence, and viral host range [13]. It was not the first time that this method was used to generate attenuated virus strains, the 17D yellow fever virus vaccine which was obtained after almost 400 passages of yellow fever virus in primary chicken and mouse cell cultures represents one of the most effective human vaccines [13].

These improved smallpox vaccines include Lister clone 16m8, MVA, and the Dairen I strain [14], all of which have been shown to be safer than their predecessors [61].

### 5.1. LC16m8

LC16m8 is an attenuated, replicating smallpox vaccine developed in Japan in 1975 as a temperature-sensitive VACV strain derived from the original Japanese Lister strain (VACV-LO) [62]. During the 1970s vaccination campaign against smallpox in Japan, over 50,000 children were vaccinated with LC16m8 [63]. VACV-LO was initially passaged 36 times in primary rabbit kidney epithelial (PRK) cells at 30 °C. Then, subclones from PRK passages were transferred to monkey kidney (Vero) cells to test their replication capacity in primate tissue [63,64] from which the LC16 strain was obtained. This strain was passaged 6 more times on PRK cells to produce LC16m0. After additional passages of LC16m0 on PRK cells, VACV strain LC16m8 was finally generated [63]. Both LC16m8 and VACV-LO form plaques on chicken chorioallantoic membranes (CAM), while LC16m8 forms smaller plaques. Being a temperature-sensitive strain, passaging of LC16m8 in PRK at 41 °C was not as efficient as for VACV-LO [64]. Furthermore, death and some adverse events like cardiac dysfunction and encephalopathy had been observed upon vaccination with VACV-LO, while these complications were absent during the vaccination campaign in Japan using LC16m8 [64,65].

#### LC16m8 as B5R Deletion Mutant

The LC16m8 strain contains a frame-shifting single nucleotide deletion of guanosine in the *B5R* gene, which encodes a protein with homology to complement regulatory proteins incorporated in extracellular enveloped viruses (EEV) [63,64]. In VACV, B5 is crucial for forming intracellular enveloped virions (IEV) in cooperation with viral proteins A33 and A36 [65,66] and induces protective neutralizing antibodies against EEV [67]. Despite the frame-shift mutation in *B5R*, LC16m8 is still able to induce protective immunity in mice and rabbits [68] indicating that there are likely other targets that can induce a protective immune response. Although LC16m8 is considered a less efficient vaccine compared to those without *B5R* mutation [13], it has been shown to be especially useful for people with preexisting immunity to other VACV strains because it is not effectively neutralized and can induce a more varied T-cell and antibody response [67].

### 5.2. Dairen I Strain

The Dairen I (DI) strain was obtained after 13 successive passages of the parenteral Dairen (DIE) strain in one-day-old chicken eggs [13,14]. Due to deletions in host-range genes *K1L* and *C7L*, this strain is not able to grow in most mammalian cells and cell lines such as BHK, RK13, and CV-1. Despite its restricted growth properties, DI shows potential as a non-replicating viral vector because it can express viral and inserted genes without showing cytopathic effects [69].

### 5.3. Modified Vaccinia Virus Ankara (MVA)

MVA was developed through attenuation of Chorioallantois Vaccinia Virus Ankara (CVA) starting in the late 1950s by serial tissue culture passages in chicken embryo fibroblasts (CEF). During the extensive passaging, CVA lost approximately 31 kbp of its genomic information, reducing its genome size to 178 kbp (GenBank: U94848.1). In addition to multiple gene fragmentations within the genome, the reduction is mainly due to six major deletions occurring in the left or right terminal ends. An example is shown for deletion III in Figure 1. The resulting virus was named MVA after the 516th passage and after further passaging was successfully used in the 1970s as a priming smallpox vaccine in a two-step vaccination program, in which no significant adverse events were reported after the administration of 120,000 MVA primary vaccine doses [70,71,72]. So far, 193 ORFs have been mapped in the MVA genome [41], although the sequence length for complete MVA genomes submitted to GenBank may vary due to the variable sequencing results for non-coding parts contained in the inverted terminal repeats on both ends of the genome. 

MVA lacks several immunomodulatory genes encoding receptors exploited by other OPXV, such as interferon gamma (IFNγ) and tumor necrosis factor (TNF). These additional mutations may be responsible for the pro-inflammatory gene expression, increased migration of immune cells, and the transduction capacity of pro-inflammatory signals during infection with this highly attenuated VACV strain [11]. Furthermore, MVA contains a deletion in the *K1L* gene which restricts its host range, rendering MVA replication-defective in some mammalian cells, such as Chinese hamster ovary cells (CHO) [73]. However, several other regulatory VACV gene sequences are still conserved within the MVA genome, such as *K3L* and *E3L* [74]. Both *K3L* and *E3L* encode an inhibitor of IFN-induced, double-stranded (ds) RNA-dependent protein kinase (PKR) [75,76]. PKR is a key component in mediating the antiviral actions of interferons (IFNs) through modulating protein phosphorylation and RNA degradation [77]. To inhibit PKR, the K3 protein mimics the PKR substrate alpha subunit of eukaryotic translation initiation factor 2 (eIF2) [78]. In addition to inhibiting PKR, the E3 protein also inhibits the activation of IFN-induced proteins [79] and early antiviral ubiquitin-like protein ISG15 [80], thus helping the virus evade early host immune responses. MVA mutants lacking *E3L* have impaired viral DNA replication in cells that have a strong type I IFN response such as CEF cells, resulting in restricted viral propagation [74,81]. Typically, poxviruses produce dsRNA late in their replication cycle [82]. However, MVA vectors designed to produce large amounts of early dsRNA may overcharge E3 and activate the antiviral properties of PKR. Interestingly, MVA infections with excessive early dsRNA expression displayed an increased cytokine and chemokine response in murine and human cells and may improve the immunogenicity of MVA [83].

MVA represents an extensively studied derivative of VACV with an excellent safety profile, and sustained immunogenicity upon MVA vaccination has been proven in various mammalian species [71,72,84,85] despite its replication deficiency in most mammalian cells such as those of human, monkey, mouse, or rabbit origin [86,87]. In these cells, MVA infection is abortive and viral progeny is hardly produced due to a block in viral morphogenesis, while viral genes are still expressed and viral proteins synthesized. This VACV strain provides a particular advantage since it allows for versatile use under conditions of biosafety level 1 including the production of heterologous proteins as a recombinant viral vector [84,85]. Additionally, the immunogenicity and protective capacity of MVA make this strain an excellent vaccine candidate to deliver heterologous antigens compared to replicating VACV [85,88]. Many preclinical and phase I/II clinical trials have demonstrated MVA’s ability to induce polyfunctional antigen-specific cellular and humoral immune responses in humans [43,89]. Nevertheless, MVA’s immunogenicity may be still improved. Deletion or replacement of functional immunomodulatory genes in the MVA genome may further enhance antigen-specific immune responses upon MVA vaccination.

## 6. Novel Viral Vector Vaccine Candidates Based on MVA with Improved Immunogenicity and Vaccine Performance

**Figure 2:**
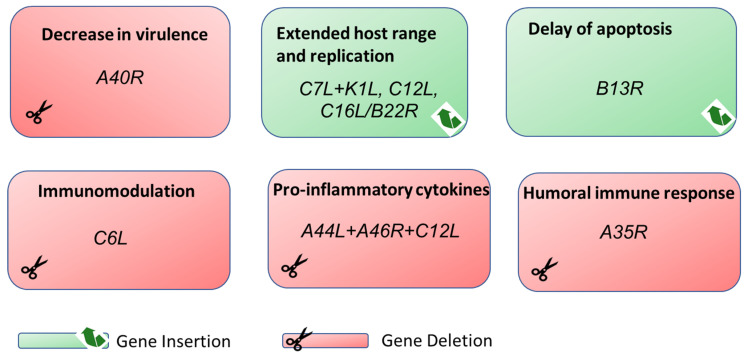
Synopsis of targeted genetic manipulations to improve immunogenicity and vaccine performance of MVA. Genetic manipulation by homologous recombination resulted in either deletion or insertion in the viral genome. Genes separated by a comma (,) have been discretely deleted or inserted. Genes separated by a plus sign (+) have been deleted or inserted in combination.

In the past, live viral vaccines were developed based on classical attenuation principles [90] that involved serial passaging of the virus in a given host [91]. In the last decades, DNA recombination techniques allowed for genomic manipulations in poxviruses such as deletion, insertion, or interruption of genes that permit targeted gene manipulation [14]. In 1982, two distinct studies demonstrated the feasibility of generating recombinant VACV which expressed the thymidine kinase gene from the herpes simplex virus [92,93]. From that point on, recombinant DNA technologies played an essential role in the sustainable development of VACV-based vaccine technology. Recently, more sophisticated innovations in biotechnology based on synthetic platforms using distinct bacterial artificial chromosome (BAC) recombination techniques, must be considered as major achievements to accelerate and to facilitate viral vector generation [94,95,96,97,98]. Additionally, recent data indicates that the insertion site of the recombinant expression cassette within the genome of MVA has an impact on the availability of the synthesized target antigens for antigen processing and presentation [99]. Here, the expression locus may influence the ability of this strain to prime optimal responses from antigens that require a direct presentation. The last generation of smallpox vaccines were developed primarily considering safety and the induction of appropriate immune responses. In this context, VACV genes with immunomodulatory and host-range-related properties are promising targets for the development of VACV-based recombinant vaccine vectors with increased and maintained immunogenicity against various pathogens [13]. In the following subsections, we will discuss the newest strategies and perspectives based on the manipulation of immunomodulatory and host-range-related genes in the MVA genome (summarized in Figure 2). We will mainly refer to the genomic nomenclature used for VACV strain Copenhagen as it is still widely spread in literature. An overview of the genetic manipulations described in this review is given in Table 2.

### 6.1. Deletion of VACV A40R Gene from MVA-B HIV-1 Vaccine Candidate Results in Increased Immunogenicity

During attenuation, CVA lost a large amount of gene coding sequences due to deletions and mutations, while the resulting MVA still retains several gene products with immunomodulatory properties. In 1999, Wilcock et al. characterized the *A40R* early gene product in VACV strain WR, which showed amino acid similarities between the A34 protein (a component of extracellular enveloped virus) and A40 [42]. In 2020, Pérez et al. compared the homologous *A40R* gene in strain Copenhagen to the MVA genome and demonstrated that it was closely related to the *MVA152R* gene which encodes an early expressed type II membrane glycoprotein most likely contributing to virulence [18]. They then generated an MVA-based HIV-1 vaccine without the *152R* gene and named it according to the Copenhagen nomenclature MVA-BΔ*A40R*. They observed enhanced polyfunctional acute and memory HIV-1-specific T-cell and humoral immune responses, indicating that A40 likely also plays a role in these functions [18].

### 6.2. Introduction of Host-Range-Related Genes into MVA Which Allow for Growth in Human Cells

As previously mentioned, MVA is incapable of assembling infectious virions in human cells [86,88]. Nevertheless, the exact molecular mechanisms of its host restriction are still not fully understood but knowledge of it could be beneficial to improve the immunogenicity or to extend the usage of MVA-based vaccines. The *C12L* gene of VACV strain Copenhagen encodes the serine protease inhibitor 1 (SPI-1). The *B22R* gene of VACV strain WR (*VACWR205*) represents the corresponding ortholog. It represents one of four known host-range-related regulatory proteins including C7/K1 and C16 which were reintroduced into recombinant host-range-extended (HRE) MVAs with rescued growth in human cells [48,110,111,112]. The deletion of the *C12L* orthologs from rabbit poxvirus (RPXV) and WR impaired replication in human and pig cell lines [106]. Inserting *C12L* into MVA resulted in increased replication by more than 100 fold in human MRC-5 cells [48]. In addition to elucidating the molecular mechanisms underlying MVA host restriction in humans, the genes mentioned before, alone or in combination, may prove highly effective in therapeutic approaches investigating the oncolytic potential of MVA or for vaccination strategies based on recombinant MVA with regained yet controllable replication potential.

### 6.3. MVA-B13R Delays Apoptosis in Antigen Presenting Cells

The VACV WR *B13R* (SPI-2) gene encodes a protein that acts as a pan-caspase inhibitor and inhibits caspase-8-mediated extrinsic apoptosis during infection [113,114]. B13 belongs to a superfamily of serine protease (serpin) inhibitors including cowpox virus cytokine response mediator A (CrmA) [107]. While at least six different VACV proteins inhibit apoptosis, a comparative study investigating the role of anti-apoptotic proteins N1, F1, B13, and Golgi apoptotic protein (GAAP), identified B13 as the most potent inhibitor both during viral infection and in the absence of infectious context [115].

MVA genes *181R/182R* are non-functional and disrupted with homology to the WR *B13R* gene [43]. Replacement of these fragmented genes in MVA with functional anti-apoptotic *B13R* (MVA-*B13R*) resulted in a significant delay of apoptosis in infected murine and human muscle cells, as well as rhesus macaque plasmacytoid dendritic cells (pDCs) and CD141+ DCs compared to wildtype MVA. MVA-*B13R* infection reduced caspase 3 activation, which was key in delaying the activation of apoptosis in muscle and antigen-presenting cells. Additionally, the antigen-specific humoral immune response upon MVA-*B13R*-based vaccination in mice was likely enhanced due to increased antigen load, persistence, and targeting to endosomal antigen processing routes [43].

### 6.4. Removal of VACV C6L Encoding a Multifunctional Inhibitor of Type I IFN Signaling in an MVA-HCV Vaccine Candidate Elicits High Immunogenicity

VACV encodes several Bcl-2-like proteins (A46, A52, B14, C1, C6, C16/B22, F1, K7, N1) that block apoptosis by binding pro-apoptotic Bcl-2 proteins such as Bak and Bax [116]. The *C6L* gene encodes C6, a multifunctional IFN antagonist. C6 acts as an inhibitor of Pattern Recognition Receptor (PRR)-induced activation of the transcription factors IRF3 and IRF7. It inhibits IRF3 and IRF7 activation downstream of TANK binding kinase 1 (TBK1) and IkB kinase-ε (IKKε), resulting in the inhibition of IFN-ß production [44,45,46]. In addition, C6 targets histone dacetylase 4 (HDAC4) for proteasomal degradation thereby interfering with STAT2-mediated type I IFN signaling [117]. 

IFN responses are vital for potent T-cell immunity mediated by MVA [118]. Deletion of VACV *C6L* from an MVA vector that expresses nearly the full-length genome of Hepatitis C virus (HCV) (MVA-HCV Δ*C6L*) could not rescue the downregulation of type I IFN expression by HCV proteins produced by MVA-HCV [47]. Both MVA-HCV and MVA-HCV Δ*C6L* induced high and polyfunctional HCV-specific CD8+ T-cell and humoral responses against HCV antigens in mice. However, they had quantitatively distinct epitope specificities where MVA-HCV favored p7 and NS2-specific responses and MVA-HCV Δ*C6L* induced higher NS3-specific CD8+ T-cell responses [47]. Since p7 and NS2 are essential for the production of infectious HCV particles [119], it is possible that the quality of the antigen-specific T-cell response could be regulated or fine-tuned by targeted manipulations of the vector backbone. 

### 6.5. Deletion of VACV A44L, A46R, and C12L Genes from the MVA Genome Improves Vector Immunogenicity and Optimizes Antigen-Specific T-Cell Responses

The *A44L* gene of strain WR encodes a 3β-hydroxysteroid dehydrogenase/Δ^5^-Δ^4^ isomerase (3β-HSD) [120], an enzyme that catalyzes the biosynthesis of steroid hormones, like glucocorticoids (GCs) [121]. The A46 protein of VACV counteracts Toll-like receptors (TLR) to inhibit the induction of pro-inflammatory cytokines [122]. Due to the anti-inflammatory effects of GCs [49] and the importance of TLRs in detecting conserved pathogen-associated molecular patterns (PAMPs), Holgado et al. (2016) deleted the *A44L-A46R* segment from the MVA genome [104] to improve immune responses. The MVA *008L* gene corresponds to *C12L* in WR and encodes an interleukin-18 binding protein that neutralizes IL-18 in humans and mice [50]. In the presence of IL-12, IL-18 activates Th1 cells and acts as a pro-inflammatory cytokine. Furthermore, IL-18 plays an important role in the stimulation process of natural killer (NK) cells and NK T cells [123]. The simultaneous deletion of *MVA008L, A44L,* and *A46R,* from MVA allowed for higher production of immune-relevant cytokines, such as IL-1β, IFN-β, IFN-γ, and IL-12 in C57BL/6 mice [104]. Similarly, deletion of the MVA *008L* ortholog in WR (*C12L* *= VACWR013*) abrogated viral interleukin-18-binding protein synthesis and reduced virulence by increasing IFN-γ production and NK and T-cell activity [49]. 

### 6.6. Deletion of A35R Increases Immunogenicity of MVA 

The poxvirus *A35R* gene is highly conserved in all sequenced mammalian-tropic poxviruses so far, including MVA [100,124]. A35 from WR suppressed MHC class II-restricted antigen presentation, chemokine, and cytokine synthesis in vitro, and decreased VACV-specific T-cell responses [101]. Infection with MVA lacking the *A35R* gene triggered increased production of virus-specific immunoglobulins in immunized BALB/c mice without significant impact on virus replication [100]. 

## 7. Conclusions

VACV has been used to combat smallpox for more than two centuries. Jenner may not have had a clear understanding of how the fluid from a cowpox lesion could protect the vaccine from smallpox, because he was not able to visualize tiny molecular compounds of virions and fathom the inner workings of viral infection as we are today. Initially, experimental use of VACV was predominantly guided by hypotheses based on simple observations in the field. Nowadays, most VACV strains have been sequenced and recombinant vectors are computer-assisted designed and synthetic viral genomes may be produced due to novel recombinant DNA technologies and other innovations in biotechnology such as BAC cloning or CRISPR/Cas9 mutagenesis. We have accumulated a massive body of knowledge and achieved many goals in regard to vaccine development. Despite the extensive choices provided by today’s technical and digital options, we should pause for a moment occasionally and reconsider our options for generating optimal future vaccines.

## Figures and Tables

**Table 1:** Homologous genes in VACV strains COP (Copenhagen), WR (Western Reserve), and MVA (Modified vaccinia virus Ankara) and their functions. Genes are named according to the nomenclature used for VACV COP [39]. Genes present in VACV WR [40] and MVA [41] are additionally indicated in the respective nomenclature which is currently used for these strains. (-) indicates absent or lost gene function.

VACV Strain	Name of Gene	Function of Gene Product	Reference
VACV COP	*A40R*	Type II membrane glycoprotein	[42]
VACV WR	*A40R (VACWR165)*	Acting as an immunomodulator	[18]
MVA	*A40R (MVA152R)*	Unknown function	[18]
VACV COP	*B13R/B14R (fragmented)*	-	
VACV WR	*B13R (VACWR195)*	Anti-apoptotic protein (Serine protease inhibitor 2 (SPI-2))	[43]
MVA	*MVA181R/182R (pseudo gene)*	Non-functional protein	[43]
VACV COP	*C6L*	Inhibition of STING dimerization and phosphorylation	[44]
VACV WR	*C6L (VACWR022)*	Suppression of type I IFN—signaling pathways	[45,46]
MVA	*C6L (MVA019L)*	Inhibition of PRR signaling	[47]
VACV COP	*C12L*	Serine protease inhibitor 1 (SPI-1)	
VACV WR	*B22R (VACWR205)*	Serine protease inhibitor 1 (SPI-1)	[48]
MVA	Not present	-	
VACV COP	Not present	-	
VACV WR	*C12L (VACWR013)*	IL-18 binding protein (host defense modulator)	[49]
MVA	*C12L (MVA008L)*	IL-18 binding protein	[50]

**Table 2:** Viral genes as targets of genetic manipulation within VACV genomes and their potential impact on vaccine immunogenicity. The “gene of interest” refers to the VACV strain Copenhagen and is depicted according to the *Hin*dIII fragment letter/ORF number nomenclature used for this strain [39]. Homologous genes for strains WR [40] and MVA [41] are named according to the current nomenclature based on a numerical taxonomy in which the ORFs are numbered consecutively starting from the left to the right end of the genome. Genetic manipulation by homologous recombination resulted in either deletion or insertion in the respective viral genome.

Gene of Interest	VACV Strain and Homologous Gene	Function of Protein	Kind of Genetic Manipulation	Impact	References
*A35R*	MVA (*146R*)	1. Suppression of MHC class II-restricted antigen presentation 2. Inhibition of virus-specific antibody production	Deletion	1. Increased production of virus-specific immunoglobulin 2. Increased numbers of virus-specific IFNγ-secreting splenocytes	[100,101]
*A36R*	WR (*VACWR159*)	1. Promotes viral egress from the host cell in association with the viral F12/E2 protein complex	Deletion	1. Viruses lacking A36 and F12/E2 proteins form smaller plaques than mutants lacking either gene alone	[102]
*A40R*	MVA (*152R*)	1. Unknown immune function	Deletion	1. Enhanced innate immune responses in infected human macrophages 2. Increased levels of IFN-β and chemokines 3. Higher memory HIV-1-specific CD4+ and CD8+ T-cell responses	[18]
*A41L*	WR (*VACWR166*)	1. Chemokine-binding protein (vCKBP)	Deletion	1. Enhanced virulence 2. Increased number of VACV-specific IFNγ-producing CD8+ T cells 3. More efficient cytotoxic T-cell responses in the spleen	[103]
*A44L*	MVA (*157L*)	1. 3β-HSD enzyme which catalyzes biosynthesis of steroid hormones	Deletion	1. Simultaneous deletion with *A46R* and *C12L (MVA008L)* induced enhanced CD4+ and CD8+ T-cell responses 2. Higher levels of IL-1β, IFN-β, IFN-γ and IL-12 3. Increased functionality and proliferative capacity of memory T-cell responses	[104]
*A46R*	MVA (*159R*)	1. Inhibition of pro-inflammatory cytokine production 2. Disruption of adaptor proteins	Deletion	1. Simultaneous deletion with *A44L* and *C12L (MVA008L)* induced enhanced CD4+ and CD8+ T-cell responses 2. Higher levels of IL-1β, IFN-β, IFN-γ and IL-12 3. Increased functionality and proliferative capacity of memory T-cell responses	[104]
*B13R*	MVA (*181R/182R* (fragmented)) WR (*VACWR195*)	1. Serine protease inhibitor 2 (SPI-2) 2. Inhibition of caspase-8-mediated extrinsic apoptosis	Insertion of functional WR gene *B13R* (*VACWR195*) into MVA	1. Delayed apoptosis in APCs	[43]
*C6L*	MVA (*019L*)	1. Blocking apoptosis by binding proapoptotic Bcl-2 proteins Bak and Bax	Deletion	1. Deletion of *C6L* in rMVA-HCV induced stronger HCV NS3-specific CD8+ T-cell responses	[47]
*C7L*	WR (*VACWR021*)	1. Host-range-related gene 2. Regulating viral cellular tropism	Deletion	1. Simultaneous deletion together with *K1L* gene results in abortive viral replication in murine and human cells	[105]
*C12L*	WR (*VACWR205*) MVA (absent)	1. Serine protease inhibitor 1 (SPI-1) (*VACWR205* corresponds to *B22R,* if the *Hin*dIII fragment letter/ORF number nomenclature was applied to WR)	Deletion from WR	1. Abortive replication in human and pig cells 2. No significant impact on replication in avian and monkey cells	[48,106,107]
			Insertion into MVA	1. Increased replication in human cells	[48]
*Absent in* *strain Copenhagen*	WR (*VACWR013*) MVA (*008L*)	1. IL-18 binding protein *(VACWR013* corresponds to *C12L,* if the *Hin*dIII fragment letter/ORF number nomenclature was applied to WR)	Deletionfrom MVA	1. Simultaneous deletion with *A44L* and *A46R* genes induced enhanced CD4+ and CD8+ T-cell responses 2. Higher levels of IL-1β, IFN-β, IFN-γ and IL-12 3. Increased functionality and proliferative capacity of memory T-cell responses	[104]
*E3L*	MVA (*050L*)	1. Encoding IFN-inducible, dsRNA-activated PKR 2. Inhibiting IFN-induced activation of IFN-stimulated genes	Deletion	1. (MVA-Δ*E3L*) is unable to replicate in CEF cells	[80,81]
*F1L*	Copenhagen	1. Inhibition of Apoptosis	Deletion	1. Enhanced cancer cell death 2. Improved safety profile 3. No impact on the replication capacity	[108]
*F12L*	WR (*VACWR051*)	1. Promotes viral egress from host cell in association with viral A36 protein	Deletion	1. Reduction in egress higher than by *A36R* deletion 2. Absence of F12 abrogates egress promoted by A36	[102]
*K1L*	WR (*VACWR032*) MVA (absent)	1. Host-range-related gene	Deletion from WR	1. Simultaneous deletion together with *C7L* gene results in abortive viral replication in murine and human cells	[105]
			Insertion into MVA	1. Recovery of K1 functions in MVA failed to extend host cell restriction to human cells	[109]
